# Differential Serum Proteomic Signatures between Acute Aortic Dissection and Acute Myocardial Infarction

**DOI:** 10.3390/biomedicines11010161

**Published:** 2023-01-08

**Authors:** You-Cian Lin, Jeen-Chen Chen, Jiunn-Min Lin, Chih-Hsiang Hsu, Ching-Feng Wu, Shao-Hsuan Kao

**Affiliations:** 1 Cardiovascular Surgery, Department of Surgery, China Medical University Hospital, Taichung 404, Taiwan; 2 Department of Medicine, China Medical University, Taichung 404, Taiwan; 3 Institute of Medicine, Chung Shan Medical University, Taichung 402, Taiwan; 4 Department of Medical Research, Chung Shan Medical University Hospital, Taichung 402, Taiwan

**Keywords:** acute aortic dissection, coronary artery disease, label-free quantitative MS/MS analysis

## Abstract

Acute aortic dissection (AAD) and acute myocardial infarction (AMI) are both severe cardiovascular diseases that may cause sudden death. However, whether serum proteins are differentially expressed between AAD and AMI remains unclear. Here, we aimed to explore serum protein profiles between AAD and AMI patients. A total of 75 serum samples were collected, including AAD patients without AMI (n = 25), AMI patients without AAD (n = 25), and normal subjects (n = 25). Protein identities and expression levels were assessed by LC-MS/MS analysis and a label-free quantitation method, respectively. After depletion of albumin and IgG, a total of 117 proteins with differential expression (fold change ≥2 or ≤−2.0, *p* < 0.05) were identified, of which 60 were upregulated and 57 were downregulated in AAD sera as compared to AMI sera. Bioinformatic analysis revealed that the differentially expressed serum proteins were mainly derived from exosomes and the extracellular space, and their molecular functions and biological processes were primarily involved in the activity of transporters and complements and the immune response. In addition, the serum level of Cadherin-5, an identified protein with significant regulation in AAD, was further evaluated by ELISA and the results showed that Cadherin-5 in AAD sera was higher that in AMI and healthy sera. Collectively, these findings reveal the differential serum protein profiles between AAD and AMI, which may reflect the divergent pathophysiological progression between the two cardiovascular diseases.

## 1. Introduction

Acute aortic dissection (AAD) is a life-threatening cardiovascular disease that was described as early as in the 18th century [1]. Several important risk factors, including long-term arterial hypertension, smoking, and dyslipidemia, have been known to result in aorta degeneration and the promotion of aortic wall fragility, which ultimately causes the structural damage of the aorta in response to blood flow and eventually leads to AAD [2,3]. According to the National Health Insurance Research Database in Taiwan, the 180-day mortality rate due to AD is as high as 22.8% [4]. Similarly, acute myocardial infarction (AMI) is also a severe cardiovascular disease that may lead to sudden death and remains the leading cause of death around the world [5]. AMI is commonly characterized by the accumulation of lipids and the resulting recruitment of inflammatory cells in the vascular endothelium [6]. Endothelium dysfunction and the inflammatory response play important roles in the process of AAD and AMI; however, whether serum protein profiles reflect the different pathophysiological progression of AAD and AMI is incompletely explored.

Serum is the fluid and solute component of blood, including abundant proteins, electrolytes, antibodies, antigens, hormones, and exogenous substances, but does not include leukocytes, erythrocytes, platelets, and clotting factors. Therefore, serum has become one of the most frequently monitored clinical samples for the evaluation of health and diseases. Among the numerous parameters in serum, the protein profile has been increasingly explored to unveil the potential biomarkers and pathophysiological features of various diseases [7,8]. In addition, the combination of rapidly advancing mass spectrometry (MS)-based proteomic methods with bioinformatic analysis has significantly accelerated the progress of clinical proteomics research. Specifically, the use of tag-label and label-free quantitative MS analysis for the protein profiling of body fluid proteomes has been clearly increasing in the past decade [9,10].

In this study, we collected serum samples from AAD patients (n = 25) and CVD patients (n = 25), and explored the protein expression profiles between patients with AAD and AMI using a label-free quantitative MS approach. Identified proteins with significant changes in serum were subjected to bioinformatic analysis to reveal the different enriched pathways between AAD and AMI.

## 2. Materials and Methods

### 2.1. Patient Enrolment

AAD patients, AMI patients, and normal subjects were consecutively enrolled from 2018 to 2019 in the Chinese Medical University Hospital (Taichung, Taiwan). For AAD and AMI patients, the time interval from the onset of chest/back/abdominal pain to admission to hospital was less than 48 h. All patients had received a complete radiological examination, including computed tomography and magnetic resonance imaging, electrocardiography (ECG), and serologic marker examination. The diagnosis of AAD followed the 2014 European guidelines and was confirmed by computed tomographic arteriography (CTA) indicating double-lumen signs. AMI was confirmed by elevated ECG and cardiac troponin T. All patients were non-smokers or had not smoked for more than 10 years. Patients with chronic kidney diseases were excluded. The protocol was approved by the Ethics Committee of the hospital (CMUH108-REC3-085) and all subjects gave informed consent for study participation. From a total of 75 subjects including AAD, AMI, and normal subjects, 10 mL blood samples were collected immediately after admission and centrifuged at 3000 rpm for 10 min at 4 °C to acquire serum. The serum was then frozen at −80 °C until further tests. The serum was thawed and immediately subjected to gel electrophoresis and in-gel digestion or ELISA assays. Repeated freeze–thaw serum was not used for analysis.

### 2.2. Label-Free Quantitative Tandem Mass Spectrometry (MS/MS) Analysis

#### 2.2.1. Protein Extraction, Gel Electrophoresis, and In-Gel Digestion

Serum samples were depleted of albumin and IgG via the ProteoPrep Blue Albumin and IgG Depletion Kit (Sigma-Aldrich, Burlington, MA, USA). The equivalent depleted sample was analyzed by 12.5% SDS-PAGE. After electrophoresis, the gels were stained with the VisPRO Protein Stain Kit (Visual Protein, Taipei City, Taiwan) and Coomassie blue stain. After staining, the gels were washed in distilled water and subsequently processed using in-gel digestion. In-gel digestion was conducted as previously described [11]. Briefly, gel lanes were cut into slices, washed three times in 25 mM ammonium bicarbonate buffer (pH 7.9), and then incubated with 50% acetonitrile in 50 mM ammonium bicarbonate buffer. Subsequently, the gel slices were reacted with 10 mM dithiothreitol for 1 h at 56 °C and alkylated with 25 mM iodoacetamide for 45 min at room temperature in the dark. After washing with the ammonium bicarbonate buffer, the gel slices were dried and incubated overnight with 20 ng/μL MS-grade Trypsin Gold (Promega, Madison, WI, USA). Peptides were extracted three times in 10 μL of 50% acetonitrile in 1% formic acid, and the pooled peptide extracts were vacuum-dried prior to LC-MS/MS analysis.

#### 2.2.2. Nano-LC Separation and MS/MS Analysis

Peptides were separated using an Ultimate System 3000 nano-LC system (Thermo Fisher Scientific, Bremen, Germany) equipped with a 75 μm ID, 25 cm length C18 Acclaim PepMap Nano-LC column (Thermo Fisher Scientific, San Jose, CA, USA) packed with 2 μm particles with a pore size of 100 Å. Mobile phase A and B were 0.1% formic acid in water and 100% acetonitrile with 0.1% formic acid, respectively. A segmented gradient from 2% to 35% solvent B in 90 min at a flow rate of 300 nL/min was conducted, and we maintained the column temperature at 35 °C for LC analysis. Intact peptide mass spectra and fragmentation spectra were acquired on a Thermo Scientific™ Orbitrap Fusion™ Lumos™ Tribrid™ Mass Spectrometer (Thermo Fisher Scientific, Hemel Hempstead, UK). Mass spectrometry (MS) analysis was performed in data-dependent mode with Full-MS (externally calibrated to a mass accuracy of <5 ppm and a resolution of 120,000 at *m*/*z* = 200), followed by high-energy collision-activated dissociation (HCD)-MS/MS of the most intense ions in 3 s. HCD-MS/MS (resolution of 15,000) was used to fragment multiply charged ions (charge state 2–7) within a 1.4 Da isolation window at a normalized collision energy of 32 eV. An AGC target at 5 × 10^5^ and 5 × 10^4^ was set for MS and MS/MS analysis, respectively, with previously selected ions dynamically excluded for 180 s. Max. injection time was 50 ms.

#### 2.2.3. Label-Free Peptide Quantification and Identification

The spectra (Thermo raw files) from tryptic digestion were loaded into Proteome Discoverer for Proteomics software, version 2.2 (Thermo Fisher Scientific, Bremen, Germany). The spectra were used to validate identification to MASCOT (www.matrixscience.com) for protein identification. All MS/MS spectra were exported from Proteome Discoverer software and searched against *Homo sapiens* (Human) in the UniProt Human database (20,365 sequences; 11,357,832 residues). The search parameters used were as follows: peptide mass tolerance set to 10 ppm, MS/MS mass tolerance set at 0.6 Da; up to two missed cleavages were allowed; cysteine carbamidomethylation was set as a fixed modification and methionine oxidation was set as a variable modification. A decoy database search was performed to determine the peptide false discovery rate (FDR) with the Target Decoy PSM Validator module. A 0.05% peptide FDR threshold was applied. Additionally, all protein identifications had MASCOT scores >25 and a high level of protein FDR confidence. The protein levels were assessed by unique peptide intensity and normalization by total peptide amount. Statistical analyses of three datasets were performed by unpaired Student’s *t*-test. All results were expressed as means with their standard deviation (SD). *p* value < 0.05 was taken as the minimum level of significance.

### 2.3. FunRich Enrichment Analysis

The LC/MS/MS data were uploaded from a Microsoft Excel spreadsheet onto FunRich software (http://www.funrich.org, accessed on 26 November 2022) [12] and Ingenuity Pathway Analysis software (https://apps.ingenuity.com, accessed on 26 November 2022). The functional enrichment analyses of serum proteins were conducted using FunRich software (version 3.1.3), which allows users to download from the FunRich database and UniProt for further analyses. A database from UniProt for human proteins (Taxon ID: 9606) was downloaded for the functional enrichment analyses of serum proteins. Options including cellular components, molecular functions, biological processes, and protein domains were used for functional enrichment analyses. Table representations of the molecular relationships between proteins were generated using Core Analysis, based on the processes showing significant associations (*p* < 0.05). All accession numbers of differentially expressed proteins (DEPs) were also examined using the STRING software (version 11.5, https://string-db.org, accessed on 26 November 2022) for convenient presentation and protein–protein interaction analysis.

### 2.4. Quantitative ELISA Analysis

The concentration of Cadherin-5 (VE-cadherin) in serum was determined with the Human VE Cadherin ELISA Kit (#ab210968, Abcam, Cambridge, UK) according to the manufacturer’s instructions. Three independent experiments were performed for statistical analysis.

### 2.5. Statistical Analysis

Data were expressed as the means ± standard error. Statistical comparisons were conducted via Student’s *t*-test, Chi-square test or one-way ANOVA were performed with the post hoc Turkey honestly significant difference test using a statistical software program (SigmaStat 3.5, Systat Software, Inc. San Jose, CA, USA). *p* < 0.05 was considered significant.

## 3. Results

### 3.1. Clinical Characterics of Subjects

A total of 75 subjects, including 25 AAD and 25 AMI patients and 25 normal subjects, were involved in the study, and their clinical characteristics are shown in Table 1. Age and gender distribution had no significant difference between the AAD, AMI, and normal groups. General risk factors including body mass index, hypertension, LDL, HDL, TC, and TG also showed insignificant differences between the AAD and the AMI groups (*p* > 0.05, Table 1). In AAD subjects, the numbers of patients with type A and type B were 12 and 13, respectively; and two AAD patients were also diagnosed with Marfan syndrome.

### 3.2. Serum Protein Profiles between AAD and AMI Patients

**Figure 1:**
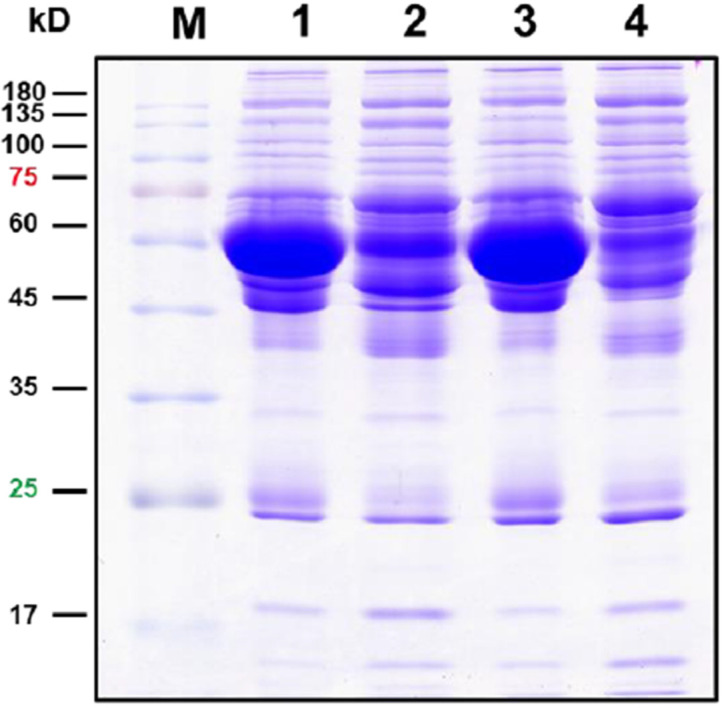
Protein profiles on SDS-PAGE. Here, 30 μg proteins were electrophoresed on 12.5% SDS-PAGE, and the proteins were visualized by Coomassie brilliant blue staining. Lane 1, original serum of AAD patients; Lane 2, depleted serum of AAD patients; Lane 3, original serum of AMI patients; Lane 2, depleted serum of AMI patients.

After the depletion of albumin and IgG, serum proteins were electrophoresed and subjected to in-gel digestion for label-free MS analysis. The serum proteins analyzed by SDS-PAGE are shown in Figure 1. A database search using the Proteome Discover software (v2.2, Thermo Fisher Scientific Inc, Bremen, Germany) was conducted, and a total of 481 proteins were identified. The protein identification was performed by screening the protein identities with at least two unique peptides, and quantitation was carried out by normalizing the intensity and determining the ratio using the relative quantitative values of the sample. The cutoff for the identification of DEPs was a *p* value < 0.05. The results showed that 422 DEPs were identified (Table 2). Among these identified DEPs, 60 DEPs with fold change ≥2.0 exhibited upregulation, while 57 DEPs with a fold change of ≤−2.0 exhibited downregulation (Table 2, Supplementary Table S1). The top 20 upregulated and downregulated DEPs are shown in Table 3.

### 3.3. Functional Enrichment Analysis of the Serum Proteins with Differential Abundance

**Figure 2:**
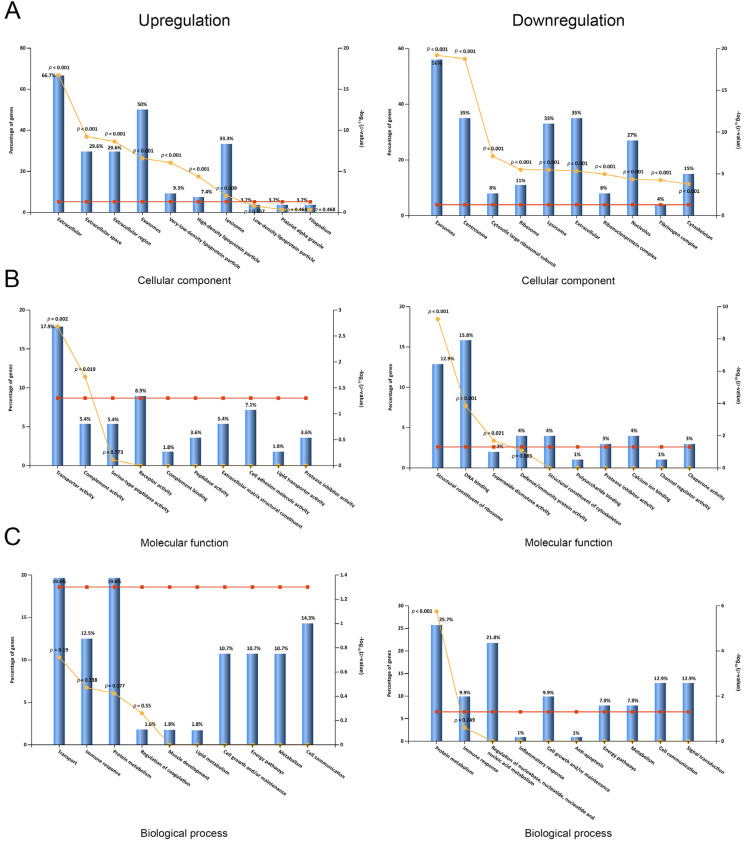
Subcellular localization and GO classification of serum DEPs. (**A**) Subcellular localization of serum proteins with upregulation and downregulation. (**B**) Molecular functions of GO classification of serum proteins with upregulation and downregulation. (**C**) Biological processes of GO classification of serum proteins with upregulation and downregulation.

Next, we explored the differential serum profiles between AAD and AMI by systematically summarizing and analyzing the DEPs and their individual functions, subcellular localization, and biological processes. Functional enrichment analysis of cellular components (CC), molecular functions (MF), and biological processes (BP) was performed via the FunRich software, as described. A summary of the CC, MF, and BP analyses is shown in Figure 1. The results of the CC analysis showed that the subcellular distribution of the upregulated DEPs was enriched in the extracellular part, exosomes, very-low-density lipoprotein particles, high-density lipoprotein particles, and lysosomes (Figure 2A, left panel). On the contrary, the subcellular distribution of downregulated DEPs was enriched in exosomes, centrosomes, ribosomes, lysosome, nuclei, the fibrinogen complex, and the cytoskeleton (Figure 2A, right panel). In the MF analysis, the upregulated DEPs were enriched in transporter activity and complement activity (Figure 1B, left panel), and the downregulated DEPs were enriched in the structural constituents of ribosomes, DNA binding, and superoxide dismutase activity (Figure 2B, right panel). In the BP analysis, the upregulated DEPs were enriched in the processes of transport, the immune response, and protein metabolism; however, the changes were not significant (Figure 2C, left panel, *p* > 0.19). In contrast to the upregulated DEPs, the downregulated DEPs were enriched in the processes of protein metabolism, the immune response, and the regulation of nucleic acid metabolism, and only enrichment in protein metabolism was significantly changed (*p* < 0.001, Figure 2C, right panel).

### 3.4. Upstream Regulator and Disease/Biofunction Analysis by Ingenuity Pathway Analysis (IPA)

**Figure 3:**
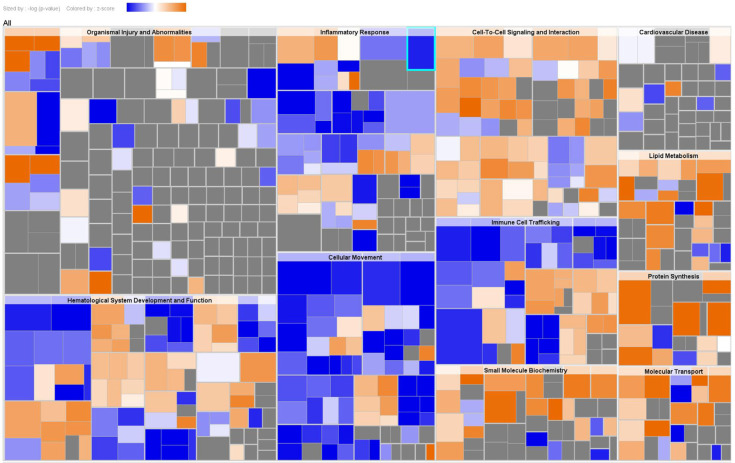
Disease and biofunction analysis of serum DEPs. A hierarchical heat map generated by disease and function analysis of serum DEPs. The main boxes represent related families of functions or categories. Each rectangle represents a specific annotation of a biological function or disease, and its color shows whether the status is predicted to be increased (orange) or decreased (blue). Higher absolute Z-scores are exhibited with darker colors. The size of the rectangles correlates with increased overlap significance (*p* value).

To explore the cascade of transcriptional regulators that may be involved in the DEPs in serum between AAD and AMI, upstream regulator analysis by IPA was conducted. The top 10 predicted transcriptional regulators of the analysis based on IPA *p* values of overlap and Z-scores are shown in Table 4. Among the predicted regulators, MYCN, IL6, MLXIPL, and STAT3 were predicted as inhibited in AAD as compared to AMI. Furthermore, disease and biofunction analysis was also performed by using IPA for significantly changed proteins in serum. The main diseases and biofunctions were predicted as upregulated or downregulated based on the IPA Z-score (Z > 2.0 or Z < −2.0). Specifically, the disease/function annotations of protein synthesis/expression, tumor necrosis, cancer cell death, and glucose metabolism disorder were predicted to be increased. On the contrary, the annotations of inflammation, angiogenesis, vasculogenesis, and endothelial cell proliferation/development were predicted to be decreased (Supplementary Table S2, disease and biofunction analysis). These annotations of DEPs were primarily categorized into organismal injury and abnormalities, hematological system development and function, inflammatory responses, cellular movement, cell–cell signaling and interaction, immune cell trafficking, and small molecule biochemistry (Figure 3).

### 3.5. Evaluation of Cadherin-5 in Serum from AAD Patients as Compared to AMI Patients and Normal Subjects

**Figure 4:**
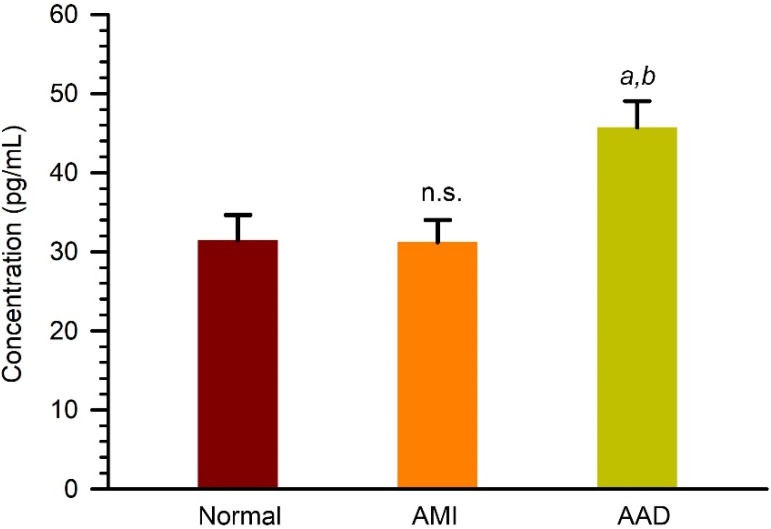
Quantitation of Cadherin-5 (VE-cadherin) in serum samples from normal, AMI, and AAD patients. Serum samples were analyzed by using a quantitative ELISA kit. *a* and *b*, *p* < 0.01 as compared to normal group; *p* < 0.005 as compared to AMI group. n.s., not significant as compare to normal group.

Among the identified serum proteins with differential expression, Cadherin-5, also known as vascular endothelial cadherin, is the major cadherin involved in homotypic cell–cell adhesion between endothelial cells and plays a central role in regulating endothelial barrier function [13]. To further evaluate the upregulation of Cadherin-5 in serum, we assessed the serum levels of Cadherin-5 in AAD, AMI, and normal subjects by using quantitative ELISA assays. As shown in Figure 4, our results showed that the concentration of Cadherin-5 was 31.5 ± 3.1 pg/mL, 31.2 ± 2.7 pg/mL, and 45.7 ± 3.2 pg/mL in AAD, AMI, and normal serum samples, respectively. These findings showed that Cadherin-5 was upregulated in AAD serum compared to AMI or normal serum.

## 4. Discussion

AAD is a severe disease with a high risk of mortality, and the annual incidence of AAD is approximately 5–30 cases per million population [12]. The prevalence of AAD and AMI has been increasing in the past decade, accompanied by the high incidence of hypertension, diabetes, and other chronic diseases [14]. In addition, the mortality rate of AAD in untreated patients after symptom onset increases by 1–2% hourly [15]. The precipitous chest pain or interscapular migrating pain is the most typical AAD syndrome; however, other sever cardiovascular disorders such as myocardial ischemia may also exhibit similar symptoms, and these clinical signs of AAD and AMI may further coexist with other conditions. Previous reports show that nearly one third of AAD patients are initially suspected as having other cardiovascular disorders, and several cases of AAD are associated with ECG signs of myocardial ischemia [12,16,17]. In these cases, the diagnosis of AAD may be delayed or even missed, and sometimes even only recorded at autopsy [16]. Accordingly, AAD can still be overlooked and misdiagnosed, and the exploration of serum protein signatures may further reveal the diverse pathophysiological development and/or progression of AAD and AMI.

Label-free quantitative MS/MS analysis is a recently developed high-throughput proteomic approach and has been widely used in exploring differential protein expression profiles in serum and plasma [15,18,19]. For AAD, several potential circulating markers, obtained by using isobaric tags for relative/absolute quantitation, have been reported previously, such as Lumican, D-dimer, CRP, and TSP-1 [20,21]. However, these potential circulating biomarkers have insufficient specificity for AAD and exhibit various expression levels in different chest diseases. Accordingly, there are still no circulating markers to reflect the physiological state of multiple pathologies. In this study, we conducted a label-free quantitative MS-based approach to comprehensively explore the differential serum proteomic signatures between AAD and AMI. Among the identified serum proteins, our label-free quantitative analyses reveal that 60 and 57 serum proteins are significantly upregulated and downregulated in AAD as compared to AMI. Further IPA analysis shows that the DEPs involved in inflammation, angiogenesis, vasculogenesis, and endothelial cell proliferation/development are predicted as significantly decreased. These differentially expressed protein signatures may help to further obtain a diagnosis of AAD, AMI, or the mixed type.

Atherosclerosis is one of the leading causes of AMI, and inflammatory mediators such as IL-6, TNFα, and CRP are potential biomarkers for the diagnosis of AMI in patients with angina [22]. The potential atherosclerotic aorta shows higher expression of IL-6 and IL-1β [23]. Recently, the CRP-to-albumin ratio was reported to be associated with the severity of AMI [24]. Consistently, our upstream regulator analysis predicts that IL-6 and STAT3, an important transcription factor for IL-1β and IL-6 production, are inhibited in AAD serum compared to AMI serum. Similarly, our analysis also reveals that inflammatory CRP is downregulated in AAD serum compared to AMI serum. In parallel, several upregulated serum proteins identified here have also been reported to be involved in various vascular disorders. For example, moesin is regarded as a biomarker of endothelial injury in sepsis, associated with increased vascular permeability [25]; mRNA expression of transgelin in aortic tissue is downregulated in AD patients compared to healthy subjects [26]; and kallistatin, a serine proteinase inhibitor, is negatively associated with the diagnosis and growth of abdominal aortic aneurysms [27]. Taken together, these findings suggest that inflammatory mediators in AAD patients are relatively lower than those in AMI; however, proteinase inhibitors and endothelial adherens junction proteins are relatively higher in AAD patients than in AMI patients.

Interestingly, our findings show that the abundance of several serum proteins, including cystatin-M (Q15828), β2-microglobulin (P61769), and leucine-rich α2-glycoprotein (P02750), is higher in AAD patients as compared to that in AMI patients, but these serum proteins are reported to show lower abundance in AAD patients compared to that in acute myocardial infarction (AMI) patients and healthy controls [18]. These discrepancies may result from the comparison between different types of CVDs (AAD/AMI vs. AAD/AMI) and may also reflect the notion that serum protein signatures are subjected to differences in the pathophysiological development and progression of CVDs. The specificity of serum protein signatures in response to different CVDs still needs further investigation.

Cadherin-5 (vascular endothelial-cadherin) is reported to play important roles in several types of aortic disorders [28,29,30]. Cadherin-5 is a member of the atypical/type II subgroup of cadherin homophilic adhesion proteins. Cadherin-5 is expressed on the surfaces of vascular endothelial cells from early on in embryogenesis through adulthood, on hematopoietic cell progenitors, and on a subpopulation of hematopoietic stem cells. In addition to cell–cell interaction, Cadherin-5 is involved in governing the proliferation, survival, cell shape, migration, and polarity of vascular endothelial cells [31]. In vascular injury, such as atherosclerotic lesions, a disintegrin and metalloprotease (ADAM) family members are upregulated and play a role in the ectodomain shedding of adhesion molecules during leukocyte recruitment, resulting in the cleavage of the Cadherin-5 ectodomain and formation of an endothelial cell gap [32]. Therefore, we suggest that the upregulated Cadherin-5 in serum may attribute to the proteolysis of membrane-bound Cadherin-5. However, further investigation is needed.

In this study, partial patients with AAD and AMI also had comorbidities, such as hypertension, mild diabetes, and hyperlipidemia, and therefore they were treated with anti-hypertensive, anti-diabetes, or anti-hyperlipidemia medications. Since these medications might change the serum protein profile, the serum DEPs identified in this study likely include these proteins.

## 5. Conclusions

This study aimed to explore the serum proteomic signatures of AAD and AMI, which exhibit some similar symptoms but result from diverse types of pathological progression. Our findings reveal differential serum protein signatures between AAD and AMI, including 60 upregulated and 57 downregulated proteins in AAD serum compared to AMI serum. By using bioinformatic analysis, these differential serum protein signatures reveal the potential pathophysiological features between AAD and AMI. These findings suggest that the differential expression signatures of proteinase inhibitors, endothelial adherent junction proteins, and inflammatory mediators may become a foundation for the development of biomarkers to distinguish AAD and AMI via further large-scale studies in the future.

## Figures and Tables

**Table 1:** Clinical characteristics of subjects.

	AAD	AMI	Normal	*p* Value
Age (mean ± SD)	58.3 ± 10.2	60.2 ± 9.6	59.6 ± 8.5	0.115 ^a^
Female (%)	11 (44%)	12 (48%)	13 (52%)	0.603 ^b^
Male (%)	14 (56%)	13 (52%)	12 (48%)	0.515 ^b^
Body mass index (kg/m^2^)	21.4 ± 2.5	20.8 ± 2.1	21.7 ± 2.1	0.056 ^a^
Hypertension (%)	25 (71.4%)	24 (68.6%)	-	0.394 ^b^
SBP (mmHg, mean ± SD)	151.2 ± 12.4	148.6 ± 13.6	108.4 ± 10.3	0.016 ^a^
DBP (mmHg, mean ± SD)	93.4 ± 8.5	96.1 ± 9.6	78.5 ± 9.3	0.021 ^a^
Type A/B (n)	12/13	n.d.	n.d.	-
Marfan syndrome (n)	2	n.d.	n.d.	-
LDL (mmol/L)	2.59 ± 0.78	2.71 ± 0.65	2.15 ± 0.36	0.032 ^a^
HDL (mmol/L)	1.19 ± 0.28	1.09 ± 0.21	1.83 ± 0.21	0.028 ^a^
TC (mmol/L)	3.89 ± 1.02	3.77 ± 0.89	3.52 ± 0.71	0.042 ^a^
TG (mmol/L)	2.13 ± 1.02	1.98 ±1.21	1.41 ± 0.21	0.021 ^a^
HbA1c (%)	6.1 ± 0.9	5.9 ± 1.0	4.4 ± 0.9	0.035 ^a^
eGFR (mL/min/1.73 m^2^)	80.5 ± 8.2	79.2 ± 8.1	82.3 ± 7.1	0.067 ^a^

^a^, One-way ANOVA; ^b^, chi-square test; n.d., not detected; SBP, systolic blood pressure; DBP, diastolic blood pressure; LDL, low-density lipoprotein; HDL, high-density lipoprotein; TC, total cholesterol; TG, triglyceride.

**Table 2:** Summary of proteins identified in serum samples from AAD and AMI.

	Identified Proteins		* Differentially Expressed Proteins	
	Upregulation	Downregulation	Upregulation	Downregulation
AAD vs. AMI	236	186	60	57

* Differentially expressed proteins had a fold change in protein level AAD/AMI ≥2.0 or ≤−2.0, determined using label-free quantitative MS/MS analysis.

**Table 3:** Identified serum proteins with significantly differential abundance (top 20 proteins with upregulation and downregulation) in AAD serum compared to that in AMI serum.

Protein ID	Protein Name (Gene)	Fold Change
P02144	Myoglobin (MB)	23.92
P26038	Moesin (MSN)	20.18
P01817	Immunoglobulin heavy variable 2–5 (IGHV2-5)	11.77
Q01995	Transgelin (TAGLN)	10.58
Q9NQ79	Cartilage acidic protein 1 (CRTAC1)	10.11
P07451	Carbonic anhydrase 3 (CA3)	9.03
P55056	Apolipoprotein C-IV (APOC4)	8.72
P01764	Immunoglobulin heavy variable 3–23 (IGHV3-23)	5.8
P33151	Cadherin-5 (CDH5)	5.53
P23083	Immunoglobulin heavy variable 1–2 (IGHV1-2)	5.12
P11021	Endoplasmic reticulum chaperone BiP (HSPA5)	4.75
P55058	Phospholipid transfer protein (PLTP)	4.73
P05154	Plasma serine protease inhibitor (SERPINA5)	4.37
Q9NTU7	Cerebellin-4 (CBLN4)	4.36
P02786	Transferrin receptor protein 1 (TFRC)	4.27
P80748	Immunoglobulin lambda variable 3–21 (IGLV3-21)	4.23
P29622	Kallistatin (SERPINA4)	4.13
P01833	Polymeric immunoglobulin receptor (PIGR)	4.08
P06727	Apolipoprotein A-IV (APOA4)	3.86
P35030	Trypsin-3 (PRSS3)	3.78
P61769	Beta-2-microglobulin (B2M)	−3.72
Q86UD1	Out at first protein homolog (OAF)	−3.87
Q02543	60S ribosomal protein L18a (RPL18A)	−4.01
Q15828	Cystatin-M (CST6)	−4.48
P02671	Fibrinogen alpha chain (FGA)	−4.84
P06310	Immunoglobulin kappa variable 2–30 (IGKV2-30)	−4.92
A0A075B6S6	Immunoglobulin kappa variable 2D-30 (IGKV2D-30)	−5.02
P59665	Neutrophil defensin 1 (DEFA1)	−5.12
P08779	Keratin, type I cytoskeletal 16 (KRT16)	−6.29
P29508	Serpin B3 (SERPINB3)	−6.3
P07477	Trypsin-1 (PRSS1)	−6.42
Q04695	Keratin, type I cytoskeletal 17 (KRT17)	−7.55
P62241	40S ribosomal protein S8 (RPS8)	−9.05
P01706	Immunoglobulin lambda variable 2–11 (IGLV2-11)	−10.87
P48668	Keratin, type II cytoskeletal 6C (KRT6C)	−12.01
P20742	Pregnancy zone protein (PZP)	−14.68
P08294	Extracellular superoxide dismutase [Cu-Zn] (SOD3)	−23.14
P02741	C-reactive protein (CRP)	−25.31
Q08830	Fibrinogen-like protein 1 (FGL1)	−36.79
P0DJI8	Serum amyloid A-1 protein (SAA1)	−165.04

Fold changes are presented as the ratio of AAD/AMI, determined using label-free quantitative MS/MS analysis. Positive or negative indicates upregulation or downregulation, respectively.

**Table 4:** Upstream regulators predicted by IPA.

Upstream Regulator	Molecule Type	Predicted Activation State	Activation Z-Score	*p* Value of Overlap
MYCN	transcription regulator	Inhibited	−2.892	1.79 × 10^−16^
IL6	cytokine	Inhibited	−3.592	2.21 × 10^−14^
MLXIPL	transcription regulator	Inhibited	−3.051	1.05 × 10^−12^
STAT3	transcription regulator	Inhibited	−2.156	6.41 × 10^−11^
MYC	transcription regulator	-	−1.846	1.61 × 10^−10^
TCR	complex	-	−1.607	3.40 × 10^−10^
JUN	transcription regulator	-	−1.634	4.68 × 10^−10^
OSM	cytokine	-	−1.815	6.70 × 10^−10^
TNF	cytokine	-	−1.941	1.99 × 10^−09^

Sorted by the statistically significant molecules.

## Data Availability

The dataset generated for this study are available on request to the corresponding author.

## Supplementary Materials

The following supporting information can be downloaded at: https://www.mdpi.com/article/10.3390/biomedicines11010161/s1, Table S1: LC/MS/MS identification of differentially expressed proteins in Homo sapiens for AAD vs. AMI; Table S2: disease and biofunction analysis.
