# Supplementary material for: Differential Serum Proteomic Signatures between Acute Aortic Dissection and Acute Myocardial Infarction
Source: Biomedicines. 2023 Jan 8;11(1):161. doi: 10.3390/biomedicines11010161 (PMC9855332; doi:10.3390/biomedicines11010161)
Supplement: Supplementary file 1 [file biomedicines-11-00161-s001.zip › Supplementary Table S1.pdf]

Supplementary Table S1. LC/MS/MS identification of differential expressed proteins in *Homo Sapiens* for AAD vs AMI (Fold Change ≧ 2.0 or ≦ -2.0)

Protein-ID	Protein Name	Intensity AMI	Intensity AAD	AAD vs CAD
P02144	Myoglobin	688397.4	16467905	23.92
P26038	Moesin	38710.67	781306.9	20.18
P01817	Immunoglobulin heavy variable 2-5	725759.8	8540282	11.77
Q01995	Transgelin	31565.22	334020	10.58
Q9NQ79	Cartilage acidic protein 1	630022.1	6366481	10.11
P07451	Carbonic anhydrase 3	327022	2952756	9.03
P55056	Apolipoprotein C-IV	813703.3	7096052	8.72
P01764	Immunoglobulin heavy variable 3-23	848560.6	4920354	5.8
P33151	Cadherin-5	989881.1	5473888	5.53
P23083	Immunoglobulin heavy variable 1-2	831486.3	4258844	5.12
P11021	Endoplasmic reticulum chaperone BiP	489251.7	2326385	4.75
P55058	Phospholipid transfer protein	749504.2	3546692	4.73
P05154	Plasma serine protease inhibitor	520705.2	2275669	4.37
Q9NTU7	Cerebellin-4	118239.9	515919.8	4.36
P02786	Transferrin receptor protein 1	547354.9	2336864	4.27
P80748	Immunoglobulin lambda variable 3-21	278021.3	1176171	4.23
P29622	Kallistatin	17151421	70888052	4.13
P01833	Polymeric immunoglobulin receptor	7376291	30080537	4.08
P06727	Apolipoprotein A-IV	6.34E+08	2.45E+09	3.86
P35030	Trypsin-3	426502.4	1612806	3.78
P04430	Immunoglobulin kappa variable 1-16	1325133	4971265	3.75
P07996	Thrombospondin-1	19853735	70848913	3.57
P20851	C4b-binding protein beta chain	6527605	22846242	3.5
P01602	Immunoglobulin kappa variable 1-5	2257924	7658749	3.39
P02649	Apolipoprotein E	1.73E+08	5.8E+08	3.36
Q9UHG3	Prenylcysteine oxidase 1	973961.3	3274979	3.36
P01594	Immunoglobulin kappa variable 1-33	676009.7	2199658	3.25
P25705	ATP synthase subunit alpha, mitochondrial	125488	397614.8	3.17
P07359	Platelet glycoprotein Ib alpha chain	1513771	4748382	3.14
P03952	Plasma kallikrein	36467427	1.1E+08	3.01
P19320	Vascular cell adhesion protein 1	486549.1	1412516	2.9
A0A0J9YX35	Immunoglobulin heavy variable 3-64D	4239158	12153428	2.87
P06396	Gelsolin	1.49E+08	4.18E+08	2.8
P02766	Transthyretin	2.63E+08	7.28E+08	2.77
P08582	Melanotransferrin	822883.6	2252613	2.74
A0A0C4DH67	Immunoglobulin kappa variable 1-8	2534803	6853676	2.7
P08603	Complement factor H	2.16E+08	5.69E+08	2.64
A0A0C4DH73	Immunoglobulin kappa variable 1-12	263538.7	689624.1	2.62
P14151	L-selectin	2932724	7630780	2.6
Q13201	Multimerin-1	771145	1973833	2.56
A0A075B6S5	Immunoglobulin kappa variable 1-27	1803679	4527945	2.51
Q96IY4	Carboxypeptidase B2	4425868	10882839	2.46
Q04756	Hepatocyte growth factor activator	11416057	27792960	2.43
P0DOX4	Immunoglobulin epsilon heavy chain	2870988	6963363	2.43
P19338	Nucleolin	248706.6	604716.4	2.43
Q96PD5	N-acetylmuramoyl-L-alanine amidase	75785136	1.81E+08	2.39
P62244	40S ribosomal protein S15a	211678.6	498223.3	2.35
A0A0C4DH35	Probable non-functional immunoglobulin heavy variable 3-35	6143242	14332570	2.33
P49747	Cartilage oligomeric matrix protein	1611038	3421079	2.12
P01859	Immunoglobulin heavy constant gamma 2	1.18E+08	2.47E+08	2.08
P69891	Hemoglobin subunit gamma-1	3515395	7302973	2.08
P02042	Hemoglobin subunit delta	48487786	1E+08	2.07
Q5BKX8	Caveolae-associated protein 4	2705650	5613971	2.07
O14791	Apolipoprotein L1	39149219	80485446	2.06
O95445	Apolipoprotein M	36433626	74924971	2.06

Protein-ID	Protein Name	Intensity AMI	Intensity AAD	AAD vs CAD
P02765	Alpha-2-HS-glycoprotein	6.93E+08	1.41E+09	2.04
P10643	Complement component C7	62713832	1.27E+08	2.03
A0A0B4J1X5	Immunoglobulin heavy variable 3-74	596594.1	1212717	2.03
P0DOX5	Immunoglobulin gamma-1 heavy chain	7.68E+08	1.55E+09	2.01
P00748	Coagulation factor XII	68560941	1.37E+08	2.01
P53999	Activated RNA polymerase II transcriptional coactivator p15	264494.1	131871.9	-2.01
P0C0S8	Histone H2A type 1	1630629	805315.8	-2.02
P25786	Proteasome subunit alpha type-1	317883.4	156975.9	-2.03
P68431	Histone H3.1	8952716	4387615	-2.04
A0A087WSX0	Immunoglobulin lambda variable 5-45	2274002	1110598	-2.05
P00338	L-lactate dehydrogenase A chain	2031686	987038.6	-2.06
P00746	Complement factor D	1282935	624018.7	-2.06
P32969	60S ribosomal protein L9	216442.1	103612.8	-2.09
P47756	F-actin-capping protein subunit beta	1059434	497245.9	-2.13
P62906	60S ribosomal protein L10a	1154523	530857.8	-2.17
P05089	Arginase-1	389594.1	178641.6	-2.18
P31151	Protein S100-A7	986671	445807.3	-2.21
P04211	Immunoglobulin lambda variable 7-43	1589517	666360.8	-2.39
P02750	Leucine-rich alpha-2-glycoprotein	3.95E+08	1.6E+08	-2.46
P0DOX8	Immunoglobulin lambda-1 light chain	2.43E+09	9.64E+08	-2.52
P20618	Proteasome subunit beta type-1	371606.1	147211.9	-2.52
P35268	60S ribosomal protein L22	738555.5	290938.6	-2.54
P49247	Ribose-5-phosphate isomerase	139728.2	54382.17	-2.57
O00299	Chloride intracellular channel protein 1	15768595	5909216	-2.67
P01614	Immunoglobulin kappa variable 2D-40	5132799	1895354	-2.71
P12429	Annexin A3	419189.5	153768.9	-2.73
O60814	Histone H2B type 1-K	3812394	1370795	-2.78
A0A075B6I0	Immunoglobulin lambda variable 8-61	25827886	8879334	-2.91
P61247	40S ribosomal protein S3a	787941.7	269652	-2.92
Q14019	Coactosin-like protein	417889.8	141684.9	-2.95
P23284	Peptidyl-prolyl cis-trans isomerase B	1332566	449064	-2.97
P42766	60S ribosomal protein L35	610791.7	205959.1	-2.97
P62701	40S ribosomal protein S4, X isoform	1315116	435110.3	-3.02
P27348	14-3-3 protein theta	1591228	507515.9	-3.14
P27105	Erythrocyte band 7 integral membrane protein	2665236	834476.5	-3.19
P61224	Ras-related protein Rap-1b	2540939	775637.7	-3.28
P04179	Superoxide dismutase [Mn], mitochondrial	973299.9	295180.8	-3.3
P02679	Fibrinogen gamma chain	1.52E+10	4.39E+09	-3.47
P02675	Fibrinogen beta chain	1.59E+10	4.41E+09	-3.61
P61353	60S ribosomal protein L27	694210.4	191914.9	-3.62
P18428	Lipopolysaccharide-binding protein	4010985	1103103	-3.64
P05451	Lithostathine-1-alpha	661492.6	178063.3	-3.71
P61769	Beta-2-microglobulin	7099637	1910219	-3.72
Q86UD1	Out at first protein homolog	419531.1	108268.1	-3.87
Q02543	60S ribosomal protein L18a	631806.8	157630	-4.01
Q15828	Cystatin-M	1100160	245431.8	-4.48
P02671	Fibrinogen alpha chain	1.64E+10	3.4E+09	-4.84
P06310	Immunoglobulin kappa variable 2-30	1701506	346056.3	-4.92
A0A075B6S6	Immunoglobulin kappa variable 2D-30	3288637	654678.6	-5.02
P59665	Neutrophil defensin 1	3279206	639860.4	-5.12
P08779	Keratin, type I cytoskeletal 16	7723039	1227207	-6.29
P29508	Serpin B3	1601955	254138	-6.3
P07477	Trypsin-1	1445146	225201.7	-6.42
Q04695	Keratin, type I cytoskeletal 17	1421981	188254	-7.55
P62241	40S ribosomal protein S8	654473.2	72339.52	-9.05
P01706	Immunoglobulin lambda variable 2-11	13413550	1233640	-10.87
P48668	Keratin, type II cytoskeletal 6C	7739265	644258.5	-12.01
P20742	Pregnancy zone protein	57507538	3916361	-14.68

Protein-ID	Protein Name	Intensity AMI	Intensity AAD	AAD vs CAD
P08294	Extracellular superoxide dismutase [Cu-Zn]	3983527	172166	-23.14
P02741	C-reactive protein	8.98E+08	35469096	-25.31
Q08830	Fibrinogen-like protein 1	3059643	83160.23	-36.79
P0DJI8	Serum amyloid A-1 protein	4932068	29883.9	-165.04
